# Do managed exchange rates and monetary sterilization encourage capital inflows?

**DOI:** 10.1371/journal.pone.0238205

**Published:** 2020-08-28

**Authors:** Vandana Arya, Tony Cavoli, Ilke Onur

**Affiliations:** UniSA Business School, University of South Australia, Adelaide, Australia; Institute for Economic Forecasting, Romanian Academy, ROMANIA

## Abstract

Economies with exchange rate pegs generally attract higher capital inflows either through lower transaction costs of trade and finance, or by encouraging investors to exploit any interest differentials, or where foreign exchange (FX) interventions are sterilized, any previous interest differentials are preserved. This paper examines these relationships using FDI, portfolio and bank inflows for 28 emerging market economies. We find that greater fixity of the exchange rate and sterilized intervention can potentially encourage capital inflows, and that the effect is magnified when combined. Further, we find that the effect differs by region, and it is larger for higher inflows.

## 1. Introduction

The prevailing intuition behind the effects of fixed or managed exchange rates on the inflow of foreign capital is generally straightforward and well recognized. First, pegged economies result in greater nominal exchange stability which will produce lower transaction costs of trade and finance, thereby encouraging cross border capital flows [1]. Second, fixed exchange rates may encourage investors to exploit even small interest differentials, thus potentially stimulating capital flows [2]. Third, during prevailing periods of high capital inflow, where FX interventions are partially or completely sterilized by monetary authorities, any interest differential existing before the inflow episode can be preserved, therefore perpetuating the capital inflow episode [3, 4].

This paper examines the relationship between exchange rate regime, sterilized intervention and capital inflows for 28 emerging market economies (EMEs) for the period 1990–2013. EMEs experience significant inflows of foreign capital such that they may induce a reserve flow. This is because EMEs are more likely to have managed exchange rate regimes [5]. This reserve flow may be fully or partially offset by monetary sterilization. We pay attention to whether the effects differ by region (Asia vs Latin America), and by composition of capital inflow (FDI, portfolio and bank). We know from [6] that regional variations exist in the composition of capital inflows and in the policy implications surrounding exchange rate regime choice and financial policy, and from [7] and others that show that capital flows themselves behave differently by composition.

We find that greater fixity does indeed encourage capital inflows and all inflow types are impacted. Further, we find significant evidence of a monetary sterilization effect; if the degree of monetary sterilization increases, then there is a resultant capital inflow. This effect is more pronounced when interacted with exchange rate regime. We also find that capital inflows are sensitive to both exchange rate regimes and sterilization, more for Asia than for Latin America. Finally, panel quantile regression estimates for the full sample indicate that both exchange rate regime and sterilization impact capital inflows at higher quantiles–suggesting that authorities are concerned about financial instability brought about by larger volumes of capital inflow.

Significant policy implications follow from this analysis. Greater exchange rate fixity can stimulate the inflow of foreign capital because it removes some of the risk faced by domestic borrowers. Moreover, monetary sterilization appears to also promote further capital inflows where sterilization policy keeps local interest rates high such that they propagate the capital inflow episode. Finally, the magnitude of the capital inflow episode matters for both exchange rate management and sterilization, suggesting that that authorities are concerned about financial instability brought about by larger volumes of capital inflow.

This paper is organized as follows: The next section presents a simple model that sets out a possible channel through which capital inflows can be impacted by monetary sterilization under fixed exchange rates. Section 3 presents the data and estimation details while section 4 discusses the results of the empirical analysis. Section 5 concludes and provides some policy messages.

## 2. Exchange rate regimes, sterilization and capital inflows

The impact of monetary sterilization policy is particularly of interest. If, say, the reserve effects of capital inflows (or similar shock) under fixed exchange rates are fully sterilized, then the effect of the pegged exchange rate regime on subsequent capital inflows is magnified by the interest rate effect of full sterilization. The analysis that follows sets out, in a simple but instructive model, the channels through which these effects may take place. Consider the following:

Δd=−λΔf (1)

Δf=ca+κ (2)

$$ca={ѳ}_{1}\Delta e-{ѳ}_{2}\hat{y}+\varepsilon$$ (3)

$$\kappa =\psi_{0}(i-i^{*})+\psi_{1}\kappa_{-1}+\psi_{2}\hat{y}$$ (4)

$$i^{*}=i^{f}+\Delta e_{+1}+\omega$$ (5)

Eq (1) is a standard sterilization equation. The sterilization coefficient, *λ* captures the extent to which authorities use domestic assets (*d*) to offset the reserve effects of balance of payments flows (*f*). Complete sterilization can be represented by *λ* = 1, while partial sterilization means 0*<λ<*1. Eq (2) is a balance of payment identity under fixed exchange rates, where the current account balance (ca) and capital flow (κ) equal the change in foreign reserves. Eq (3) represents the current account, a function of the exchange rate, *e*, and domestic income, *ŷ*, as well as the last term (*ε*) which is an exogenous current account shock. Eq (4) is an expression for capital flows where *i* (i*) represents the return on domestic (foreign) assets respectively. Eq (4) represents capital flows as a function of an interest sensitive component (the uncovered interest deviation, i–i*), a persistence term as captured by lagged capital flows, *κ*_*-1*_, and a pro-cyclical effect. It is tempting to perhaps specify Eq (4) to capture the components of capital inflow. The current specification keeps the model tractable but still manages to convey the idea that capital inflow source matters to ascertain the effects of sterilization.

Eq (5) is an expression for the domestic currency return on foreign financial assets along with a global liquidity shock (*ω*). Money supply and demand are given, respectively, by the following:

Δm=Δd+Δf (6)

$$\Delta m^{d}=-\alpha \Delta i+\gamma \hat{y}+\delta \Delta p$$ (7)

We note here that Eq (6) holds by approximation. Equalising Δ*m*^*d*^ and Δ*m*, substituting for i*, *κ*, ca, Δ*d* and Δ*f*, and rearranging, we obtain:

$$i=\Phi [(1-\lambda )\psi_{0}(i^{f}+\Delta e_{+1}+\omega )-(1-\lambda )({ѳ}_{1}\Delta e–{ѳ}_{2}\hat{y}+\varepsilon +\psi_{1}\kappa_{-1}+\psi_{2}\hat{y})+\delta \Delta p+\gamma \hat{y}+\alpha i_{-1}]$$ (8)

where: $\Phi =\left(\frac{1}{\propto +(1-\lambda )\psi_{0}}\right)$.

To examine the extent to which an exogenous shock (in this instance to the current account) impacts the domestic interest rate (and hence capital inflows), consider the effect of a shock on the domestic interest rate as follows:

$$\frac{\partial i}{\partial \varepsilon }=-\frac{\left(1-\lambda \right)}{\propto +(1-\lambda )\psi_{0}}<0$$ (9)

This holds for partial sterilization (0<λ<1). In other words, a reserve inflow under fixed exchange rate that is partially sterilized will place downward pressure on the interest rate. It is easy to see that, for full sterilization (λ = 1), $\frac{\partial i}{\partial \varepsilon }=0$. As such, if the reserve effects of a current account shock are fully offset, then the local interest rate remains at existing, pre-shock levels. Importantly, to the extent that capital inflows are interest sensitive, complete sterilization can potentially induce a capital inflow episode. In the case of a positive reserve flow, sterilization will reduce the stock of high powered money and raise domestic interest rates through a liquidity effect. With local rates at or close to pre-inflow levels, an environment is created that encourages capital inflows. It is possible that the preservation of high interest rates after a current account shock may not be attributable to sterilization. However, it has been observed empirically that in both Asia and Latin America, the degree of sterilization is high, and in some cases, very close to unity. As such, sterilization remains a viable explanation [3] considers the case of a capital inflow surge and examines a scenario where sterilization preserves the local interest at the levels that existed prior to the surge. Using an interest rate determination model, he shows that sterilization could perpetuate the capital inflow surge by keeping the interest rate high, but the direct effect of the inflow of capital was not tested. In the sections below, we directly test the relationship between sterilization, exchange rate regime on capital inflows.

## 3. Data and estimation

The empirical strategy for this paper is to estimate the effect of exchange rate fixity on capital inflows and to assess whether interest rates that are kept high through sterilization stimulated these inflows. We employ data from 28 EMEs for 1990–2013 and estimate the following:

$$CF_{it}=\beta_{1}ERREGIME_{it}+\beta_{2}STER_{it}+\beta_{3}\prime CONTROLS_{it}+\delta_{i}+\mu_{t}+\varepsilon_{it}$$ (10)

where CF refers to the inflow of foreign capital (as % of GDP). We examine three main components of capital inflow; FDI, Portfolio and Bank inflows. *ERREGIME* is the degree of exchange rate fixity for each country and year. We employ two broad measures of exchange rate regime classification. The first one (*ERR*) is based on the coarse classification of the exchange rate regime measure introduced by [8]. This measure ranges between no separate tender/pre-announced peg (coded as 1) to freely falling (coded as 5). In order to express the variable as a measure of exchange rate fixity–so that we are consistent with ERINT (Eq 11)–we subtract the raw data from 6 –so that a freely floating regime is recorded as ERR = 1 and a pre-announced peg is ERR = 5. Data are available for download through the Quarterly Journal of Economics Dataverse. See [8] for details. The second measure (*ERINT*) is an exchange rate intervention measure based around the concept of exchange market pressure (see [9] for a recent account) and is measured as follows:

$$ERINT=\frac{│\Delta ft│}{\left(│\Delta et│+│\Delta ft│\right)}$$ (11)

where, ∆*e*_*t*_ is the change in the exchange rate (local currency per USD), and ∆*f*_*t*_ is the change in net foreign assets (scaled by lagged reserve money). The index is constructed to return a value between zero (exchange rates flexible) to one (exchange rates fixed). Exchange market pressure (EMP) measures are based on the notion that changes occurring in foreign exchange markets are either observed directly through exchange rate changes, or that exchange rates themselves do not move, but are otherwise observed in changes in reserves or interest rates. The seminal contribution here is [10]. The authors employ a simple EMP measure that subtracts reserves changes from currency changes. The measure used in this paper (used in [11, 12] among others) is a more nuanced version of the basic EMP measure with more desirable properties. This makes it easier to interpret while retaining the simplicity of the basic measure. For robustness, we also employed Δ*f* as a measure of FX intervention, as well as a version of Eq (11) using nominal effective exchange rates (NEER). The results from these are not presented in the next section as they are broadly similar to the results using Eq (10). They are available on request.

The effect of sterilization is captured by an autoregressive distributed lag (ARDL) version of Eq (1) as follows:

$$\Delta d_{t}=\tau +\lambda_{t}\Delta f_{t}+\theta (L)\Delta d_{t-1}+\varphi (L)\Delta f_{t-1}+\omega_{t}$$ (12)

Eq (12) is estimated for each country using a rolling regression estimator with a window size of 60 months and a step size of one month (note that For Mainland China and Hong Kong, we used a 48-month window due to the truncated sample available for those countries). The model is run for each country individually. The 12-monthly average absolute value of the time-varying sterilization coefficient (*λ*_*t*_) is estimated and utilized as *STER*_*it*_ in Eq (10). The vector of controls includes inflation, GDP, interest rate differentials, trade openness, institutional quality, a GFC dummy, financial liberalization (Chinn-Ito Index) and financial development (Details of countries sampled, variables used, as well as descriptive statistics are available in S1 Appendix).

Our baseline specification is the panel least squares model. If managed exchange rates stimulate capital inflows, we would expect to see *β*_*1*_>0. A fixed exchange rate allows policymakers to sterilize capital inflows, thus preventing local interest from adjusting downwards. Hence, an increase in the extent of sterilization would expand capital inflows. As such, *β*_*2*_>0 would be expected. In addition to examining the effect over the three main components of capital inflow, we also investigate whether any regional differences (Asia vs Latin America) exist. The sample contains countries that possess very high levels of capital inflow (for example, Korea), as well as those with very low levels (for example, Panama). To assess whether the effects of FX and sterilized intervention vary by the magnitude of capital inflow, we also use a panel quantile regression model. We note some further details regarding estimation. Both country and time fixed effects are modelled. The employment of a panel least squares (LS) model implies that we estimate each component of capital inflows in isolation, as 3 separate equations. As part of our robustness testing, we also estimated a Seemingly Unrelated Regression (SUR) model to capture any cross equation residual correlations. The results were substantively similar to our baseline OLS results and are consequently not reported here, but they are available upon request. Further, acknowledging that capital inflows are related (see [6]), we include those components that are not the dependent variable as regressors to capture these interactions.

## 4. Results and discussion

Tables 1 and 2 present the results of the panel least squares (LS) estimates for the individual components of capital inflow–FDI, portfolio and bank inflows. We have two specifications for each regression. Specification 2 includes an interaction term between the exchange rate regime and sterilization variables. Specification 1, however, does not include the interaction term. The effect of the measure of exchange rate regime (*ERR*) on capital inflows is shown in Table 1 and is positive and statistically significant for all components and specifications except for specification 1 in FDI. The effect of FX intervention (*ERINT*) on capital flows is presented in Table 2 and is positive and significant across all components and specifications except for specification 1 for Bank inflows. This suggests that intervention involving foreign reserves to promote exchange rate fixity is possibly effective in encouraging further capital inflows. The effect of *STER* on capital inflows is also robustly positive and significant except for specification 1 in Bank, both in Tables 1 and 2. This suggests, as detailed above, that the extent of sterilization can act to further propagate the capital inflow episode by maintaining local interest rates at pre-inflow levels. We also take the opportunity to re-run the regressions using the first difference of the sterilization coefficient, Δ*f*. The effects of ERR and ERINT, as well as Δ*f* are very similar to the results in Table 1 (results are available on request).

**Table 1:** Sterilization, exchange rate regimes (ERR) and capital inflows: Full sample dependent variable—Capital flows (FDI, portfolio and bank flows).

	FDI		Portfolio		Bank	
***ERR***	0.140	1.890***	0.419*	1.550**	1.422**	0.098*
	(0.192)	(0.558)	(0.203)	(0.606)	(0.589)	(0.014)
***STER***	0.820**	1.173***	0.823**	2.479**	0.669	1.216**
	(0.461)	(0.421)	(0.487)	(0.937)	(1.426)	(0.715)
ERR*STER		2.339***		0.998*		1.751**
		(0.605)		(0.660)		(0.929)
**FDI**			0.343***	0.349***	0.335**	0.359**
			(0.047)	(0.048)	(0.144)	(0.146)
**Portfolio**	0.307***	0.303***			0.761***	0.756***
	(0.042)	(0.041)			(0.132)	(0.133)
**Bank**	0.035**	0.036**	0.089***	0.088***		
	(0.015)	(0.015)	(0.015)	(0.016)		
**GDPPC**	0.000***	0.000***	-0.000*	-0.000*	0.000***	0.000***
	(0.000)	(0.000)	(0.000)	(0.000)	(0.000)	(0.000)
**Inflation**	-0.008	-0.011	-0.024	-0.023	0.083	0.085
	(0.018)	(0.017)	(0.019)	(0.019)	(0.054)	(0.055)
**TO**	0.034***	0.034***	-0.026***	-0.026***	0.129***	0.128***
	(0.007)	(0.006)	(0.007)	(0.007)	(0.020)	(0.020)
**DCBGDP**	0.005	0.007	0.004	0.004	-0.065**	-0.066***
	(0.008)	(0.008)	(0.009)	(0.009)	(0.025)	(0.025)
**GovtExp**	0.007	-0.053	0.081	0.094	-0.235	-0.189
	(0.081)	(0.081)	(0.085)	(0.087)	(0.250)	(0.255)
**IQ**	-0.030	-0.052*	0.051*	0.056*	0.139	0.155*
	(0.029)	(0.029)	(0.031)	(0.032)	(0.091)	(0.092)
**FinLib**	0.317**	0.379***	-0.158	-0.173	0.116	0.062
	(0.132)	(0.131)	(0.140)	(0.142)	(0.411)	(0.416)
**GFC**	0.142	0.078	-1.190***	-1.176***	-1.454	-1.407
	(0.339)	(0.334)	(0.354)	(0.354)	(1.045)	(1.047)
**IRD**	0.010	0.005	-0.026*	-0.025*	0.054	0.057
	(0.013)	(0.013)	(0.014)	(0.014)	(0.040)	(0.040)
**_cons**	1.539	-4.447	-0.912	0.352	-5.227	-2.735
	(2.375)	(2.805)	(2.511)	(3.020)	(5.252)	(2.779)
**N** **R-sq**	494	494	494	494	494	494
	0.304	0.327	0.239	0.240	0.265	0.266

The symbols*, ** and *** indicate statistical significance at the 10%, 5% and 1% levels, respectively. Standard errors in parentheses.

**Table 2:** Sterilization, intervention (*ERINT*) and capital Inflows: Full sample dependent variable–capital flows (FDI, portfolio and bank flows).

	FDI		Portfolio		Bank	
**ERINT**	0.823**	1.272**	0.913*	0.090**	-1.969	1.176*
	(0.453)	(0.624)	(0.475)	(0.009)	(1.402)	(0.820)
**STER**	1.201**	1.234**	0.879**	0.367*	0.372	1.585**
	(0.461)	(0.482)	(0.484)	(0.049)	(1.429)	(0.982)
ERINT*STER		1.655*		1.190*		2.622**
		(0.602)		(0.688)		(0.978)
**FDI**			0.342***	0.344***	0.322**	0.325**
			(0.047)	(0.047)	(0.145)	(0.145)
**Portfolio**	0.309***	0.310***			0.779***	0.776***
	(0.042)	(0.042)			(0.133)	(0.134)
**Bank**	0.033**	0.034**	0.090***	0.089***		
	(0.015)	(0.015)	(0.015)	(0.015)		
**GDPPC**	0.000***	0.000***	-0.000	-0.000	0.000**	0.000**
	(0.000)	(0.000)	(0.000)	(0.000)	(0.000)	(0.000)
**Inflation**	-0.003	-0.003	-0.016	-0.016	0.020	0.020
	(0.016)	(0.016)	(0.017)	(0.017)	(0.049)	(0.049)
**TO**	0.034***	0.034***	-0.027***	-0.027***	0.133***	0.133***
	(0.007)	(0.007)	(0.007)	(0.007)	(0.020)	(0.020)
**DCBGDP**	0.005	0.004	0.004	0.005	-0.060**	-0.060**
	(0.008)	(0.008)	(0.009)	(0.009)	(0.025)	(0.025)
**GovtExp**	0.003	0.002	0.094	0.094	-0.248	-0.246
	(0.081)	(0.081)	(0.085)	(0.085)	(0.252)	(0.252)
**IQ**	-0.032	-0.031	0.046	0.045	0.172*	0.170*
	(0.029)	(0.029)	(0.031)	(0.031)	(0.090)	(0.090)
**FinLib**	0.324**	0.349***	-0.173	-0.191	0.111	0.072
	(0.133)	(0.135)	(0.140)	(0.143)	(0.414)	(0.421)
**GFC**	0.143	0.137	-1.158***	-1.153***	-1.557	-1.547
	(0.339)	(0.339)	(0.353)	(0.353)	(1.050)	(1.051)
**IRD**	0.010	0.010	-0.028**	-0.029**	0.061	0.059
	(0.013)	(0.013)	(0.014)	(0.014)	(0.041)	(0.041)
**_cons**	1.207	0.141	-1.816	-1.050	-6.202	-8.505
	(2.340)	(2.558)	(2.462)	(2.692)	(7.200)	(7.893)
**N**	493	493	493	493	493	493
**R-sq**	0.304	0.306	0.244	0.245	0.259	0.260

The symbols*, ** and *** indicate statistical significance at the 10%, 5% and 1% levels, respectively. Standard errors in parentheses.

We also attempt to capture the effect of sterilization given the extent of exchange rate fixity through interaction terms (specification 2). We see that the interaction terms are all consistently positive and significant. This suggests that the cumulative effect of a fixed exchange rate and sterilization has a magnified impact on capital inflows.

Next, we focus our attention to the emerging market economies in Asia and Latin America separately. Table 3 presents a summary of the key estimates for the Asian sample. These results generally mirror the estimates for the full sample in that the effects of *ERR* and *ERINT* are positive, as are the signs for *STER*. The coefficients, though, are not as strongly significant. As with the full sample estimates, we present results for the interaction terms for regime and sterilization. There is some evidence of a magnification effect, but it is not as emphatic as those for the full sample. Table 4 presents the results for the Latin American sample. There is evidence of positive effects throughout, and there is evidence of a magnification effect through a higher coefficient value for the interaction term. However, these results are not as robustly significant as for Asia, or the full sample.

**Table 3:** Summary of key estimates: Asian sample dependent variable–capital flows (FDI, portfolio and bank flows).

Panel A: Using *ERR*						
	FDI		Portfolio		Bank	
***ERR***	0.058	3.887***	0.640**	.476**	1.71	1.271
	(0.34)	(1.229)	(0.386)	(0.667)	(1.188)	(4.546)
***STER***	1.239	1.202***	1.771	6.821	1.153*	0.795
	(0.995)	(0.333)	(1.13)	(6.389)	(0.507)	(0.808)
ERR*STER		4.088***		1.214*		1.468**
		(1.264)		(0.511)		(0.679)
Panel B: Using *ERINT*						
***ERINT***	1.401	1.796*	1.833*	0.784*	0.956*	1.198*
	(0.857)	(0.762)	(0.973)	(0.213)	(0.367)	(0.661)
***STER***	1.692*	3.621**	0.672*	0.603*	0.919*	1.832*
	(0.988)	(1.941)	(0.121)	(0.207)	(0.522)	(0.69)
ERINT*STER		0.763*		1.118*		1.295**
		(0.27)		(0.64)		(0.37)

The symbols*, ** and *** indicate statistical significance at the 10%, 5% and 1% levels, respectively. Standard errors in parentheses. The fixed effects model is estimated with the following control variables: inflation, GDP per capita, interest rate differentials, trade openness, government expenditure, institutional quality, a GFC dummy, financial lliberalization and financial development. The results for these variables are omitted for brevity, but they are available upon request.

**Table 4:** Summary of key estimates: Latin American sample dependent variable–capital flows (FDI, portfolio and bank flows).

Panel A: Using *ERR*						
	FDI		Portfolio		Bank	
***ERR***	0.288	1.575***	0.28	-0.366	1.111**	1.011
	(0.218)	(0.585)	(0.179)	(0.497)	(0.466)	(1.301)
***STER***	0.076	2.024***	0.101	3.015	0.411	1.608*
	(0.486)	(0.661)	(0.4)	(2.275)	(1.049)	(0.935)
ERR*STER		2.321***		0.808		2.645*
		(0.679)		(0.581)		(1.516)
Panel B: Using *ERINT*						
***ERINT***	0.874*	2.763**	0.882**	0.407	0.834	3.227
	(0.477)	(1.294)	(0.39)	(1.077)	(1.047)	(2.856)
***STER***	0.11	1.771	0.08	0.336	0.561	2.658
	(0.485)	(1.164)	(0.398)	(0.964)	(1.059)	(2.557)
ERINT*ST		2.414		0.604		3.049
		(1.539)		(1.275)		(3.384)

The symbols*, ** and *** indicate statistical significance at the 10%, 5% and 1% levels, respectively. Standard errors in parentheses. The fixed effects model is estimated with the following control variables: inflation, GDP, interest rate differentials, trade openness, government expenditure, institutional quality, a GFC dummy, financial liberalization and financial development. The results for these variables are omitted for brevity, but they are available upon request.

Finally, we run a panel quantile model to mirror the panel LS estimates generated above. These results are presented in Table 5 –for the full sample only. The quantile regression estimates do exhibit a bit of variation. Regarding *STER*, whether *ERR* or *ERINT* is utilized (although the effect appears more pronounced for *ERINT*), we observe more statistical significance for our main variables of interest at lower quantiles when FDI is considered. For Bank and Portfolio inflows, however, we observe the significant variables at higher quantiles. This suggests that policymakers are concerned about the possible adverse effects of greater inflow volumes for those capital inflows that are relatively more reversible (Bank and Portfolio) and are thus likely to induce financial instability (see [7]). This may induce central banks to provide proportionally greater FX intervention, and consequently sterilization in order to mitigate the reserve effects of that intervention.

**Table 5:** Quantile regressions. Summary of key estimates: full sample.

Panel A: Using *ERR*										
**Dependent Variable–FDI**										
	**10th**		**25th**		**50th**		**75th**		**90th**	
***ERR***	0.239*	0.926	0.042	1.592***	0.09	0.97	0.339	1.380*	0.742*	2.088
	(0.137)	(0.586)	(0.176)	(0.548)	(0.291)	(0.799)	(0.228)	(0.807)	(0.439)	(1.35)
***STER***	1.946***	3.269*	1.675***	1.197**	1.437***	2.638**	0.411	4.165	0.277	1.486
	(0.341)	(1.808)	(0.345)	(0.613)	(0.468)	(1.187)	(0.549)	(3.225)	(0.865)	(6.101)
ERR*STER		1.263**		1.968***		1.029*		-1.157		1.478
		(0.606)		(0.687)		(0.64)		(0.849)		(1.491)
**Dependent Variable–Portfolio**										
	**10th**		**25th**		**50th**		**75th**		**90th**	
***ERR***	0.254*	0.071	0.255***	0.221	0.307**	0.620**	0.406***	0.560*	0.017	0.801
	(0.13)	(0.462)	(0.093)	(0.236)	(0.128)	(0.304)	(0.104)	(0.286)	(0.3)	(0.804)
***STER***	0.079	1.7	0.254	0.906	0.056*	1.254*	0.366	1.149*	0.377	3.363
	(0.253)	(2.277)	(0.198)	(1.097)	(0.016)	(0.645)	(0.509)	(0.565)	(0.997)	(4.119)
ERR*STER		0.451		0.291		0.492*		0.513*		0.957
		(0.543)		(0.255)		(0.286)		(0.201)		(1.037)
**Dependent Variable–Bank**										
	**10th**		**25th**		**50th**		**75th**		**90th**	
***ERR***	0.035	0.753	0.719***	1.121	0.749***	0.794	1.120***	0.792*	1.482***	1.502**
	(0.588)	(1.679)	(0.22)	(0.75)	(0.235)	(0.637)	(0.366)	(0.337)	(0.546)	(0.73)
***STER***	1.575	1.019	0.087	1.556	0.16	0.214	0.729	1.144*	3.381***	3.416
	(2.364)	(8.312)	(0.735)	(3.487)	(0.467)	(2.794)	(0.782)	(0.85)	(1.245)	(6.131)
ERR*STER		0.754		0.447		0.089		0.488*		0.024
		(2.059)		(0.88)		(0.651)		(0.172)		(1.391)
Panel B: Using *ERINT*										
**Dependent Variable–FDI**										
	**10th**		**25th**		**50th**		**75th**		**90th**	
***ERINT***	-0.334	-0.341	0.086	1.649	0.054	0.099**	0.158*	1.480*	0.205	2.36
	(0.264)	(1.224)	(0.236)	(1.399)	(0.562)	(1.744)	(0.047)	(0.529)	(0.91)	(2.005)
***STER***	-1.939	-1.959	1.672***	3.330***	1.524**	1.689**	0.496*	0.671	0.534	1.811
	(0.476)	(1.026)	(0.362)	(1.156)	(0.636)	(1.024)	(0.135)	(1.472)	(0.98)	(1.463)
ERINT*STER		0.025		2.115		0.255*		1.736		2.409
		(1.583)		(1.595)		(0.105)		(1.669)		(2.142)
**Dependent Variable–Portfolio**										
	**10th**		**25th**		**50th**		**75th**		**90th**	
***ERINT***	0.346**	0.206	0.289**	0.032	0.010***	0.417***	1.102	0.904**	0.005	0.162
	(0.159)	(0.266)	(0.127)	(0.287)	(0.004)	(0.151)	(0.776)	(0.406)	(0.011)	(0.965)
***STER***	0.271*	0.303*	0.291**	0.214**	0.337**	0.417***	0.897**	0.382*	0.307	0.215
	(0.159)	(0.17)	(0.128)	(0.084)	(0.038)	(0.151)	(0.355)	(0.196)	(0.403)	(0.443)
ERINT*STER		0.291**		0.499**		0.772**		0.133*		0.6
		(0.147)		(0.215)		(0.416)		(0.04)		(1.22)
**Dependent Variable–Bank**										
	**10th**		**25th**		**50th**		**75th**		**90th**	
***ERINT***	-1.244	-2.744	0.114	0.027	0.621	0.921	0.021*	2.031**	0.805	1.519
	(1.263)	(2.821)	(0.627)	(2.198)	(0.473)	(1.419)	(0.007)	(1.195)	(1.358)	(1.249)
***STER***	-1.935	-2.473	0.99	1.098	0.553**	1.081**	0.042*	1.786**	2.437**	0.713
	(1.553)	(2.281)	(0.872)	(1.655)	(0.177)	(0.635)	(0.004)	(0.569)	(1.003)	(3.041)
ERINT*STER		5.307		0.103		2.077*		2.426**		2.03
		(3.558)		(2.303)		(1.354)		(0.723)		(2.179)

The symbols*, ** and *** indicate statistical significance at the 10%, 5% and 1% levels, respectively. Standard errors in parentheses. The quantile model is estimated with the following control variables: inflation, GDP, interest rate differentials, trade openness, government expenditure, institutional quality, a GFC dummy, financial liberalization and financial development. The results for these variables are omitted for brevity, but they are available upon request.

These results are notable for a number of reasons. The effect of exchange rate fixity on capital inflows is generally positive across the three inflow components. This suggests that management of the exchange rate can potentially encourage the inflow of foreign capital. This is consistent with the prevailing literature and our initial analysis suggesting that exchange rate stability removes some of the risk for investors, thus making it conducive for further capital flows. Exchange rate pegs produce less volatile exchange rates, lowering transaction costs, and encouraging cross border capital flows.

The impact of the degree of sterilized intervention is also notable. The results suggest that sterilization positively impacts capital inflows. High to complete sterilization can act to further perpetuate the inflow of foreign capital by keeping local interest rates high. This follows closely from the predictions of the model in section 2. To the extent that capital inflows are interest sensitive, and care about the risk adjusted return on capital, the process of sterilizing the reserve effects of a balance of payments imbalance (such as a current account shock) will restore interest rates to pre-inflow levels and induce further capital inflow.

We also observe a positive coefficient for the interaction term for exchange rate fixity and sterilization–indicating that the two channels through which capital inflows are being affected can appear to work in tandem. The model above provides an explanation for this; both a fixed exchange rate *and* subsequent sterilized intervention can impact capital flows. The fixed exchange rate creates a reserve flow through the process of central bank intervention in foreign exchange markets. This intervention creates an environment supportive of capital inflow, as mentioned above. Sterilization allows the reserve flow to be offset, and thus decreases the stock of high powered money and raises domestic interest rates, which also encourages capital inflows.

## 5. Conclusion and policy implications

Using data for 28 emerging market economies (EMEs) in Asia and Latin America for the period 1990–2014 we find that greater exchange rate fixity does indeed encourage capital inflows. We show that FDI, portfolio and bank inflows are generally equally sensitive to exchange rate regimes. Further, and in accordance with the results of our simple analytical framework, we observe significant evidence of a possible high/full sterilization effect where higher levels of sterilized intervention further promoted the inflow of capital. We also find that some regional differences exist; capital inflows are generally more sensitive for Asia than for Latin America. Finally, panel quantile regression estimates for the full sample indicate that both exchange rate regime and sterilization affect the more volatile components of capital inflow at higher quantiles–suggesting that authorities are concerned about financial instability brought about by larger volumes of capital inflow.

There are significant other policy implications for this analysis. Greater exchange rate fixity promotes the inflow of foreign capital because it removes some of the risk faced by domestic borrowers. Authorities need to be mindful, however, of the potential instances of adverse selection and moral hazard that exist here; exchange rate stability brought about by fixity may encourage high risk borrowers to engage in foreign borrowing, as well as existing borrowers being incentivized to engage in more risky behaviour (see [13]), potentially undermining financial stability as a result.

The policy of monetary sterilization appears to also promote further capital inflows–we conjecture that this is due to the interest sensitive nature of capital flows (perhaps indirectly observed in FDI) where sterilization policy keeps local interest rates high such that this propagates the capital inflow episode. This is, in itself a source of possible moral hazard as high interest rates attract higher inflows which may become unsustainable and increase the risk of reversals and capital flight (for more information, see [14, 15] for examples of capital flow problems for Latin America and Asia, respectively). These implications present warnings to central banks and policymakers about the need to monitor the progress of cross border financial flows and to ensure that financial stability and macroeconomic balances are not undermined by a regime of monetary sterilization under managed exchange rates.

## Supporting information

S1 File (XLSX)

S1 Appendix (DOCX)
