# Supplementary material for: Do managed exchange rates and monetary sterilization encourage capital inflows?
Source: PLoS One. 2020 Aug 28;15(8):e0238205. doi: 10.1371/journal.pone.0238205 (PMC7454974; doi:10.1371/journal.pone.0238205)
Supplement: S1 Appendix — (DOCX) [file pone.0238205.s002.docx]

**Table A1.  Data details.**

| **Variable** | **Description** | **Source** |
| --- | --- | --- |
| Foreign Direct Investment (FDI) | FDI liabilities of reporting country measured as percentage of GDP in US dollars | IFS |
| Portfolio Flows (Portfolio) | Sum of portfolio equity and debt equity liabilities of reporting country measured as percentage of GDP in US dollars. | IFS |
| Other Flows (Bank) | Bank and other liabilities of reporting country measured as percentage of GDP in US dollars. | IFS |
| Sterilization (STER) | Generated from Net foreign Assets and Net Domestic Assets data in National Currency as per eq. (12) | IFS |
| ERR | Exchange rate regime classification measure from Ilzetzki et al. (2019). See footnote 6. | Ilzetzki et al. (2019) |
| ERINT | Constructed from Net Foreign Assets and nominal exchange rate data as per eq. (11) | IFS |
| Institutional Quality (IQ) | Composite Risk Index - The weighted average of political, financial and economic risks. The highest overall rating (theoretically 100) indicates the lowest risk, and the lowest rating (theoretically zero) indicates the highest risk. | ICRG Index PRS Group, International Country Risk Guide. |
| Real GDP per capita (GDPPC) | GDP at market prices (constant 2010 US dollar) | World Bank, WDI |
| Interest Rate Differential (IRD) | The difference in domestic real interest rate and real interest rate in U.S. | IFS |
| Government Expenditure (GovtExp) | Government final expenditure as a percentage of GDP | World Bank WDI |
| Inflation | Year on year percentage change in Consumer Price Index | World Bank WDI |
| GFC | Dummy variable returning 1 for 2008-09, zero otherwise. | Authors’ calculation |
| Trade Openness (TO) | Sum of imports and exports as a ratio of real GDP | World Bank, WDI |
| Financial Development (DCBGDP) | The ratio of domestic credit to private sector provided by banks to real GDP | World Bank, WDI |
| Financial Liberalizsation (FinLib) | Chinn-Ito Index | http://web.pdx.edu/~ito/Chinn-Ito_website.htm |

Countries sampled: Asia. Bangladesh, China PR, Hong Kong, India, Indonesia, Korea, Malaysia, Pakistan, Philippines, Singapore, Sri Lanka, Thailand, Vietnam. Latin America: Argentina, Bolivia, Brazil, Chile, Colombia, Costa Rica, Dominican Rep, Guatemala, Honduras, Mexico, Nicaragua, Paraguay, Peru, Uruguay, Venezuela

**Table A2. Descriptive statistics.**

| **Full Sample** | | | | | | | | | | | |
| --- | --- | --- | --- | --- | --- | --- | --- | --- | --- | --- | --- |
| **Variable** | **Obs** | | **Mean** | | **Std. Dev.** | | **Min** | | **Max** | | |
| Foreign Direct Investment (FDI) | 744 | | 3.555914 | | 4.683715 | | -2.76 | | 38.68 | | |
| Portfolio Flows (Portfolio) | 744 | | 0.950914 | | 3.382179 | | -30.46 | | 38.12 | | |
| Other Flows (Bank) | 744 | | 1.390457 | | 9.281764 | | -89.69 | | 92.57 | | |
| Sterilization (STER) | 546 | | 0.830241 | | 0.267344 | | 0.029167 | | 1.7525 | | |
| Institutional Quality (IQ) | 744 | | 67.00536 | | 9.408917 | | 35.75 | | 91.11 | | |
| Real GDP per capita (GDPPC) | 720 | | 6466.216 | | 7386.365 | | 399.4839 | | 56029.19 | | |
| Interest Rate Differential (IRD) | 658 | | 4.942325 | | 14.1705 | | -103.7 | | 90.82 | | |
| Inflation | 743 | | 35.01 | | 325.5045 | | -4.02 | | 7481.66 | | |
| Government Expenditure (GE) | 742 | | 11.23794 | | 3.509946 | | 2.98 | | 43.48 | | |
| Trade Openness (TO) | 742 | | 86.686 | | 79.62233 | | 13.75 | | 455.28 | | |
| Financial Development (DCBGDP) | 740 | | 48.10682 | | 38.8351 | | 7.07 | | 218.12 | | |
| Financial Liberalization (FinLib) | 744 | | 0.410457 | | 1.430189 | | -1.89 | | 2.39 | | |
| **Asia** | | | | | | | | | | |  |
| **Variable** | | **Obs** | | **Mean** | | **Std. Dev.** | | **Min** | | **Max** |  |
| Foreign Direct Investment (FDI) | | 312 | | 4.053686 | | 6.517883 | | -2.76 | | 38.68 |  |
| Portfolio Flows (Portfolio) | | 312 | | 1.222019 | | 3.883575 | | -9.13 | | 35.78 |  |
| Other Flows (Bank) | | 312 | | 2.266538 | | 12.05135 | | -89.69 | | 92.57 |  |
| Sterilization (STER) | | 246 | | 0.875952 | | 0.236898 | | 0.049167 | | 1.34083 |  |
| Institutional Quality (IQ) | | 312 | | 67.44478 | | 11.71074 | | 35.75 | | 91.11 |  |
| Real GDP per capita (GDPPC) | | 312 | | 7293.351 | | 10434.63 | | 399.484 | | 56029.2 |  |
| Interest Rate Differential (IRD) | | 272 | | 0.444412 | | 4.402375 | | -31.79 | | 12.62 |  |
| Inflation | | 311 | | 6.50814 | | 7.31723 | | -4.02 | | 81.82 |  |
| Government Expenditure (GE) | | 312 | | 10.28064 | | 2.822795 | | 4.05 | | 17.61 |  |
| Trade Openness (TO) | | 312 | | 117.6023 | | 109.1311 | | 15.24 | | 455.28 |  |
| Financial Development (DCBGDP) | | 308 | | 70.38867 | | 47.37066 | | 8.8 | | 218.12 |  |
| Financial Liberalization (FinLib) | | 312 | | 0.000385 | | 1.353065 | | -1.89 | | 2.39 |  |
| **Latin America** | | | | | | | | | | |  |
| **Variable** | | **Obs** | | **Mean** | | **Std. Dev.** | | **Min** | | **Max** |  |
| Foreign Direct Investment (FDI) | | 432 | | 3.196412 | | 2.617999 | | -2.5 | | 17.13 |  |
| Portfolio Flows (Portfolio) | | 432 | | 0.755116 | | 2.957249 | | -30.46 | | 38.12 |  |
| Other Flows (Bank) | | 432 | | 0.757732 | | 6.539148 | | -39.94 | | 59.95 |  |
| Sterilization (STER) | | 300 | | 0.792758 | | 0.284891 | | 0.029167 | | 1.7525 |  |
| Institutional Quality (IQ) | | 432 | | 66.68801 | | 7.308471 | | 37.88 | | 82.38 |  |
| Real GDP per capita (GDPPC) | | 408 | | 5833.701 | | 3501.025 | | 1063.43 | | 14652.2 |  |
| Interest Rate Differential (IRD) | | 386 | | 8.111839 | | 17.45457 | | -103.7 | | 90.82 |  |
| Inflation | | 432 | | 55.5448 | | 425.863 | | -1.17 | | 7481.66 |  |
| Government Expenditure (GE) | | 430 | | 11.93252 | | 3.788532 | | 2.98 | | 43.48 |  |
| Trade Openness (TO) | | 430 | | 64.25367 | | 33.42353 | | 13.75 | | 198.77 |  |
| Financial Development (DCBGDP) | | 432 | | 32.22069 | | 19.48842 | | 7.07 | | 133.08 |  |
| Financial Liberalization (FinLib) | | 432 | | 0.70662 | | 1.412597 | | -1.89 | | 2.39 |  |

Source: Authors’ calculations
